# A multi-site repeated prevalence study of medicine shortages in community pharmacies

**DOI:** 10.1080/20523211.2024.2421271

**Published:** 2024-11-07

**Authors:** John C. Hayden, Siobhan Byrne, Chloe Cullen, Eadoin Lennon, France Pruteanu, Judith D. Strawbridge

**Affiliations:** School of Pharmacy and Biomolecular Sciences, RCSI University of Medicine and Health Sciences, Dublin, Ireland

**Keywords:** Medicine shortages, community pharmacy, Ireland, occupational stress, role expansion

## Abstract

**Background:**

Medicine shortages are a global problem. Prior studies have focused on hospitals, and staff views, with less information on community practice. This study aimed to estimate the prevalence of medicine shortages in community pharmacies and potential impact on patients.

**Method:**

Four community pharmacies (two urban, two rural) in Ireland recorded details of prescription request shortages per items dispensed. Data were gathered one study day per month from February to April 2023. A prevalence across sites was estimated and trends examined using a Poisson regression.

**Results:**

There were 76 medicine requests defined as shortages out of 3734 prescription item requests, giving a mean shortage prevalence of 2% (95% CI 1.6–2.5%). There was a non-significant, 17%, increase in shortage rate across the study period (*p* = 0.256). Higher rates were observed in the two urban pharmacies. In total, 61/76 (80%) of shortages were associated with a delay in patient treatment.

**Conclusion:**

Shortages are prevalent in community pharmacy and cause delays in patient treatment and increase in workload of pharmacy staff. Regulatory initiatives to address the issue at a manufacturer level have been proposed, although workforce planning, resourcing and professional role expansion are also required to protect pharmacy staff and patient outcomes.

## Introduction

The Covid-19 pandemic was associated with a transformation volume and type of work faced by community pharmacists. Reduced availability of other community and hospital-based healthcare, uncertainty around changing healthcare information, growing public frustration and the roll-out of national vaccination created a significant burden of leadership on community pharmacists (O'Donnell et al., 2024). Overwhelming workloads, reduced staffing and increasing complex presentations added to the significant demands on pharmacists to maintain adequate pharmaceutical care to patients. The pandemic also struck at a time when medicines supply chains were already vulnerable, resulting in medicines shortages that not only arose from pre-existing supply-related issues but also from increased demand and stockpiling attempts. While medicine shortages are not a new phenomenon, they have received growing media attention during the Covid-19 pandemic, with apparent epidemic levels of shortages experience (Campbell, 2022; Coyle, 2022; *The New York Times*, 2023).

Medicine shortages are recognised as a complex global problem by the World Health Organization (WHO) (Gray & Manasse Jr, 2012). According to the WHO definition, a medicine shortage is an insufficiency in the supply of medicines, health products, and vaccines identified by the health system as essential to meet public health and patient needs (World Health Organization, 2017). The causes of medicine shortages are multifactorial, with supply chain disruption, shortages in raw materials, general inflation, geopolitical factors, business decisions, and increased seasonal demand for medicines cited as critical factors (Azure Pharma, 2023). These reasons combine with the characteristics of supply systems to worsen any interruption in manufacturing. Notably, there is poor availability and quality of data on actual demand; inadequate management practices in procurement and the supply chain, combined with large tender contracts solely emphasising procurement at the lowest prices (World Health Organisation, 2015). Shortages can be mitigated against using appropriate risk management systems. According to Tang (2006), supply chain risk management (SCRM) is ‘the management of supply chain risks through co-ordination or collaboration among the supply chain partners so as to ensure continuity’ (Bastani et al., 2021; Tang, 2006). Broadly defined as efforts to identify, assess, mitigate, and respond to risks, SCRM refers to ‘supply chain solutions that ensure supply continues to meet the demand in case of a disruption or soon after the occurrence of such a disruption' (Vann Yaroson et al., 2024).

Medicine shortages affect many – they pose risks for patient health as a result of non-treatment, under-treatment, and possible medication errors from attempts to substitute missing medicines, they risk treatment delays, additional costs, also adverse drug reactions from unnecessary substitutions (Fox et al., 2014). Health outcomes may be compromised leading to poor disease management and increased dissatisfaction amongst patients (Batista et al., 2019; Phuong et al., 2019; Romano et al., 2022). For pharmacists and prescribers, they absorb significant work time in their management. For community pharmacists dealing with product sourcing and patient communication daily, the increased workload can contribute to occupational stress at a time of already high levels of professional burnout(Dee et al., 2023), and divert time away from direct patient care. Overall, shortages place avoidable pressure on primary care and must be addressed (McCartney, 2015).

Research on shortages to date have largely focussed on the impact of medicine shortages in a hospital setting, or are based on characterising shortage database lists (Patel et al., 2022; Ravela et al., 2022), or staff views on the issue (Griffith et al., 2012; Miljković, Batista, et al., 2020; Miljković, Godman, et al., 2020; Said et al., 2018). These studies together confirm shortages are a worsening problem, are particularly problematic in high risk clinical areas like oncology and anaesthetics, and place increased stress and workload on healthcare staff. The prevalence and impact of shortages on day-to-day community practices is less understood, yet is of significant importance to patients and the profession. This study seeks to address this gap at a community pharmacy level by estimating the prevalence of medicine shortages at repeated time points in the community pharmacy setting in Ireland, associated trends, and potential impact on patients.

## Materials and methods

This study was a repeated point-prevalence, cross-sectional, observational study set in four community pharmacies in the Republic of Ireland in spring 2023. Data were gathered in four separate sites (Pharmacy 1 [an independent rural pharmacy], Pharmacy 2 [a small chain urban pharmacy], Pharmacy 3 [an urban large chain pharmacy], and Pharmacy 4 [a rural large chain pharmacy]) between 9 am and 6 pm on agreed days for 3 consecutive months; 28 February, 23 March, and 27 April 2023.

Medicine shortages were defined in line with both the WHO and the Health Products Regulatory Authority (HPRA) of Ireland (the national Competent Authority which is responsible for co-ordinating the response to and lessening the impact of medicine shortages for relevant stakeholders) definition. They define the term medicine shortage as ‘A medicinal product shortage occurs where the supply of a medicinal product is inadequate to meet the needs of patients' (Health Products Regulatory Authority, 2023). In line with HPRA guidance, switching to alternative brands of the same active ingredient, although requiring extra work and education, was not considered a shortage. Delayed supply of medicines due to logistical reasons, or due to sourcing from an alternative supplier were also not considered shortages (as a product was actually available). Only licensed medicines were eligible for consideration in this prevalence study, and not medical devices or ancillary supplies supplied by community pharmacies. For quality control purposes, a project leadership group assessed and documented case-by-case considerations of shortages that did not fit within clear regulatory examples.

On each study day, the dispensary team was made aware of the procedure and contributed to the collection of prescription items requested that were in short supply. From 9 am to 6 pm on data capture days, a Microsoft Excel data log was kept in the community pharmacy dispensary to note all shortages identified by dispensary staff on prescription items requested. The following details were noted for each recorded shortage on the customised excel document: (1) medicine short, (2) strength, (3) active, (4) formulation, (5) reason for shortage, (6) ATC code, (7) expected duration, (8) if a delay in treatment occurred, (10) mitigation measure, (11) actions required. At the end of each study day, the data log was reviewed for accuracy by a member of the study team and each entry was checked against the study inclusion/exclusion criteria. Product Information was checked to determine if products were manufactured within Europe, and also whether the manufacturer was an innovator company or a generic product company.

A pilot day was conducted prior to data collection to ensure feasibility of the study procedures. Pilot data were not included in final results, but used to inform the determination of sufficient sample size for the study. An a-priori sample size calculation found having a sample of 1543 prescription item requests surveyed would give a margin of error of 1% with an associated 95% confidence interval assuming a prevalence estimate of 5% (based on the pilot data). Study data were then transferred to STATA® V.17 software for analysis. A Poisson regression was conducted to determine trends across months and across pharmacies (urban versus rural) in shortage prevalence, with output reported as incident risk ratios. *p* values below 0.05 were considered statistically significant. Summary data were reported as means with 95% confidence intervals.

Ethical approval was not required for this project as no human or animal data was gathered or processed, and the units of study were solely requested medicine items. The exemption from research ethics committee approval was confirmed with consultation with a Chair of a research ethics committee. Local supervising pharmacists gave permission for the study to be conducted within their pharmacies.

## Results

Across all three study days at the four study sites, there were 76 medicine requests defined as shortages out of a total of 3734 prescription item requests, giving a mean shortage prevalence of 2% (95% CI 1.6–2.5%). The mean shortage prevalence in February was 1.6% (95% CI 1–2.4%), in March it was 2.5% (95% CI 1.6–3.6%) and in April it was 2.2% (95% CI 1.4–3.2%). A Poisson regression indicated a non-significant 17% increase in shortage rate across the study period (*p* = 0.256). The overall mean rate per pharmacy varied from 1.4% to 3.2%, with higher rates observed in the two urban pharmacies with a 43% reduced rate of shortages in rural versus urban pharmacies (*p* = 0.015). In total, 61/76 (80%) of shortages were associated with a delay in patient treatment. All 76 products were manufactured at European sites according to their product information. Fifty one of 76 products were manufactured by generic product companies.

Reasons for shortages were available on the HPRA website for 54 (71%) of the shortage episodes. Where a reason was known, 39 (72%) of shortages were due to a manufacturing issue, e.g. delays, quality issues, supply chain issues, and 15 (28%) due to increased demand for the product. Based on the Anatomical Therapeutic Chemical (ATC) classification system, Class N medicines for the nervous system accounted for the most shortages at 25%, followed by class A, medicines for the alimentary system, which includes drugs for use in diabetes, anti-emetics, etc. at 20% followed by dermatologicals at 17%. During this study, Ozempic® (Semaglutide) Solution for injection pre-filled pens alone accounted for over 10% of all shortages. Topical betamethasone preparations were the second most common medicinal product shortage, accounting for 7% of total shortages. Of the 76 shortages identified, 33 (43%) related to oral tablets or capsules, 17 (22%) were topical creams/ointments/gels, and 11 were parenteral medicines (14%) (Table 1).

**Table 1.** Characteristic of products experiencing shortages (*N* = 76).

ATC	Classification	*N*	%	Formulation Type	*N*	%	Manufacturer Type	*N*	%
N	Nervous System	19	25	Oral Tablet/Capsule	33	43	Innovator Manufacturer	25	33
A	Alimentary Tract and Metabolism	15	20	Cream/Ointment/Gel	17	22	Generic Company	51	67
D	Dermatologicals	13	17	Injectable	11	14			
S	Sensory Organs	7	9	Eye Drop/Gel	6	8			
C	Cardiovascular System	6	8	Oral Liquid	5	7			
J	Anti-infective for Systemic Use	5	7	Inhaler	1	1			
R	Respiratory System	4	5	Nasal Spray	1	1			
M	Musculo-Skeletal System	3	4	Patch	1	1			
G	Genito Urinary System and Sex Hormones	2	3	Vaginal Tablet	1	1			
L	Antineoplastic and Immunomodulating Agents	1	1						
H	Systemic Hormonal Preparations, Excl. Sex Hormones and Insulins	1	1						

## Discussion

### Statement of key findings

Shortages are prevalent in daily community pharmacy and affecting patient care. Each day, a community pharmacy dispensing 400 items faces, on average, eight shortages where no product is available to supply to patients during this study period. Six of these eight shortages cause delays in treatment to patients. Additional workload and clinical risk are associated with each shortage. Temporal trends in shortages are observed which should be monitored longitudinally, and supports for the profession offered to resource and provide the necessary clinical autonomy to manage continuity of supply. As most shortages result from supply side issues with pharmaceutical manufacturers, regulatory initiatives are also necessary.

### Comparison to the literature

Various causes can be attributed to medicine shortages, with 50% of cases due to manufacturing delays, captured in the HPRA’s 2-year review of its medicine shortage framework (HPRA, 2020). Other causes include supply chain disruption and shipping delays, varied seasonal or unexpected increases in demand, geopolitical factors such as COVID-19 or political unrest. Just over a quarter of the shortages in this study, where a reason was known, were due to increased product demands, highlighting it is problems at the supply level that are the main contributor to shortages in community practice. The European Commission has recently communicated plans seeking to address the problems of shortages within the European Union (EU) (European Commission, 2023). It recognises the delocalisation of manufacture of Active Pharmaceutical Ingredients (API) for critical medicines to a limited number of non-EU locations, labour shortages and changing demographics as pertinent contributing factors to the issue of shortages. In the short term, closer national authority co-ordination is proposed, reform of pharmaceutical legislation to create a single European market. A ‘Voluntary Solidarity Mechanism for medicines’ sharing scheme is being launched, a union level of critical medicines is being defined which will facilitate subsequent risk minimisation strategies, and new Information Technology initiatives to forecast and monitor shortages are also planned. Procurement mechanisms are also discussed to maximise reliability of supply. Longer term plans include diversification of the supply chain, improving capacity and skills development within the sector. Planned legislative reform includes an early alert system and incentives for continuous supply. Interestingly, while manufacturer incentives are proposed, regulatory penalties are not and should be considered. The majority of shortages are the result of poor supply chain governance on the manufacturer side. Regulatory success in the area of air passenger rights associated with financial cost to the airlines has seen a reduction in delays and improved service quality on regulated routes versus non-regulated routes, and serves as a useful comparator (Gnutzmann & Spiewanowski, 2018).

The increasing workload associated with shortages at a community pharmacist level also highlights the need for maximising the scope of practice of pharmacists. The COVID-19 pandemic emphasised the integral role that pharmacists play as healthcare professionals in maintaining continuity of supply of medicines during times of stress on the healthcare system (Hayden & Parkin, 2020). Generic substitution rights allowing the pharmacist to supply an alternate strength, quantity, or formulation of specific products and therapeutic interchange rights allowing changes to alternative active ingredients, without requiring prior approval from a prescriber are in place in some countries, although requiring recognition of pharmacists clinical judgement and autonomy to be successful (Alsufyani et al., 2023; Chiu et al., 2022). Autonomous pharmacists prescribing rights combined with placement of pharmacists in general practice settings have also been shown to helpful in managing shortages and freeing up other healthcare provider time for direct patient care (Johnson et al., 2022). Although only four pharmacies were included in this study, we noted a decreased shortage rate in rural pharmacies. This may be due to a higher number of nearby pharmacies in urban locations allowing patients to try multiple pharmacies for unavailable items as wholesaler supplies would be consistent across settings. A Portuguese survey found rural areas were more impacted by shortages due to having an older population and lower socio-economic status (Romano et al., 2022). Regional breakdown of shortage prevalence is complex and worthy of further exploration in larger studies to tease out these issues further.

As shortages are a problem that will likely persist for years to come, the impact on pharmacist workload, and its association with professional wellbeing must also be considered. Each shortage causes additional workload and directs time away from direct pharmacy patient care. Leadership bodies and pharmacy regulators should continue to seek to identify mechanisms of resourcing pharmacy appropriate to workload, and reducing and streamlining alternative tasks associated with administrative and regulatory burden on the day-to-day practice of community pharmacy.

### Strengths and weaknesses

A strength of this research is the multi-site prospective study design increasing the generalisability of the study results. The large study sample of screened prescription requests increased the precision of the prevalence estimate. Although the study is strengthened by data collection across three consecutive months to estimate trends in shortage prevalence, having only three months included is a limitation. For example, the study period did not include a period of time of significant antibiotic shortages due to winter illnesses that preceded the study collection period. As a result, there are likely times during the year where actual prevalence rates of shortages are higher than observed in this study. This study also adopted the strict definition of shortages adopted by regulatory bodies, and a limitation is it does not capture the prevalence of and associated workload involved in occasions where brand switching is required, notably the resultant procurement efforts and education efforts required to maintain patient supply. There was a risk of under-reporting shortages. A pharmacy team consists of multiple team members where all are expected to deal with many different patients. Efforts to reduce this risk by standardising data capture days and hours, and data collection was carried out on days when a study team member was present in the community pharmacy dispensary.

### Further research

Medicines shortage prevalence in community practice should be tracked longitudinally to assess both the burden on pharmacy staff, and the potential impact of promised initiatives to address the issue. Longitudinal studies could identify both chronic gaps in supply chains at a product level, but also at a pharmacy type level (e.g. urban versus rural, chain versus independent) allowing remediation efforts to be made at a service level, beyond existing regulatory initiatives. Seasonal and temporal trends could also be identified to allow signals for provision of step up supports for community pharmacies. While our study relied on observers to capture episodes of shortages, there is a risk of missing data with this approach, and future studies could examine the potential for automation in reporting. Mechanisms by which pharmacists and pharmacy technicians may have their current professional scopes of practice expanded to minimise the professional and patient impacts of shortages must also be a priority.

## Conclusion

The study highlights that community pharmacy staff are faced with managing multiple medicines shortages each day. These shortages are impacting patient care with the majority causing delays in treatment. Each shortage requires additional workload in efforts to procure alternative products and communicate with patients and prescribers, taking pharmacy staff away from other aspects of care. While regulatory initiatives to address the issue at a manufacturer have been proposed, efforts to enhance the functionality and protect the occupational wellbeing of pharmacy staff must be equally addressed, as the problem will likely persist for the years to come.
